# Deep-learning-based natural-language-processing models to identify cardiovascular disease hospitalisations of patients with diabetes from routine visits’ text

**DOI:** 10.1038/s41598-023-45115-1

**Published:** 2023-11-05

**Authors:** Alessandro Guazzo, Enrico Longato, Gian Paolo Fadini, Mario Luca Morieri, Giovanni Sparacino, Barbara Di Camillo

**Affiliations:** 1 Department of Information Engineering, University of Padova, 35131 Padua, Italy; 2 Department of Medicine, University of Padova, Padua, Italy; 3 Department of Comparative Biomedicine and Food Science, University of Padova, Legnaro, Italy

**Keywords:** Biomedical engineering, Diabetes complications

## Abstract

Writing notes is the most widespread method to report clinical events. Therefore, most of the information about the disease history of a patient remains locked behind free-form text. Natural language processing (NLP) provides a solution to automatically transform free-form text into structured data. In the present work, electronic healthcare records data of patients with diabetes were used to develop deep-learning based NLP models to automatically identify, within free-form text describing routine visits, the occurrence of hospitalisations related to cardiovascular disease (CVDs), an outcome of diabetes. Four possible time windows of increasing level of expected difficulty were considered: infinite, 24 months, 12 months, and 6 months. Model performance was evaluated by means of the area under the precision recall curve, as well as precision, recall, and F1-score after thresholding. Results showed that the proposed NLP approach was successful for both the infinite and 24-month windows, while, as expected, performance deteriorated with shorter time windows. Possible clinical applications of tools based on the proposed NLP approach include the retrospective filling of medical records with respect to a patient’s CVD history for epidemiological and research purposes as well as for clinical decision making.

## Introduction

Diabetes is a chronic disease characterised by elevated blood glucose levels. According to data collected in 2017, 6.28% of the world population had diabetes^1^ and by 2030 its global prevalence is projected to increase to 10.1%^2^. Diabetic complications, among which cardiovascular diseases (CVDs) are the most relevant^3^, are estimated to contribute to one in nine deaths among adults aged 20–79 years, making it the ninth leading cause of death^4^. In order to delay, mitigate, or avoid diabetes-related complications, patients need to be tightly monitored by general practitioners or endocrinologists through periodic routine visits^5^. This longitudinal (from a data-flow perspective) nature of diabetes care leads to the need of describing the course of the disease over time, usually via a very large and long-lasting stream of heterogeneous data, typically handled by digital systems, such as electronic health records (EHR).

However, as for many other clinical situations, most of the information about patient history in EHR systems is locked behind free-form text^6^ as writing down notes remains the most expressive method to record clinical events^7^. As a consequence, unstructured clinical notes dominate over structured data^8,9^ and, in order to obtain datasets that can be processed by automatic algorithms, relevant information must typically be extracted via manual review by experts, thus leading to scalability and cost issues^10^.

To automatically transform the free-form text of routine visits into structured clinical data that can be further re-used and re-purposed^11^, natural language processing (NLP) models have been proposed. To mention a couple of examples, Sterling et al.^12^ used neural network regression models to predict emergency-department patient-disposition from triage notes. These algorithms were proved to be able to convert the free-form text of a triage note written by receptionist nurses into a specific patient-disposition outcome. In another article, Guan et al.^13^ compared machine learning (ML) and deep learning (DL) algorithms to identify genomic-related treatment changes reported in routine-visit progress notes of cancer patients. In the diabetes field, previous research mainly focused on the identification of the disease itself^14^ as well as some of its complications such as foot ulcer^15^, vision loss^16^, and hypoglycaemia occurrence^17^. However, to the best of our knowledge, the use of EHR data and NLP for CVD detection has not yet been thoroughly studied.

In the present study DL-based NLP models were developed to automatically identify CVD hospitalisations from the unstructured free-form text of patients’ routine visits. To search a previous hospitalisation starting from a given visit, four possible time windows corresponding to as many clinically relevant scenarios are considered. In the first scenario, hospitalisations are searched back in time to be associated with a visit without any time constraint (i.e., the hospitalisation may have occurred at any time before the considered visit). This situation might be of interest when one wants to retrospectively fill patients’ medical records with respect to CVD history using the NLP model instead of assigning personnel to read all free-form text for each patient individually. In the second scenario, hospitalisations are searched in a 24 months’ time window back in time starting from the date of the visit. Such a time window may be useful for the conduction of retrospective population studies^18^. In addition, knowing the recent CVD status enables physicians taking correct decision with regards, e.g. to medications for the management of diabetes or to pursue strict secondary preventive strategies^19^. The third scenario is identical to the second one but is expected to be much more challenging because a previous CVD hospitalisation should fall within 12 months before the visit. Finally, the fourth scenario is even more extreme as it considers only hospitalisation occurred within 6 months before the visit’s date. Using this last time window would be necessary, for instance, if one wanted to create time-to-event datasets to be later used to develop predictive models of CVD hospitalisations^20^. These scenarios are ordered by their expected complexity. Specifically, with longer time windows more hospitalisations can be found and, as a result, data become more populated and descriptive. Instead, as the time window narrows, less hospitalisations can be associated with the visits, resulting in a loss of information due to time-windowing and temporal resolution constraints imposed by the consequent domain of application. Taking this into consideration, NLP algorithms may work better in some scenarios than others and the main aim of this study is to understand which are the clinical settings of interest in which NLP approaches can be reliably used to extract structured information from unstructured medical notes. Moreover, the discrimination performance of models proposed for each scenario is assessed after implementing two different thresholding schemes (one innovative that allows for a certain degree of classification uncertainty) in two alternative settings: a natural by-visit setting, where each visit is considered independently of all the others, and a by-patient setting, where visits are aggregated with the aim of distinguishing between patients with and without a previous history of CVD hospitalisations.

## Materials and methods

### Data

The database used in this study was a typical EHR-type database collected at the Diabetic Outpatient Clinic of the University Hospital of Padova (Italy). This database contained, among other information, the free-form text of the 197,411 routine visits undergone by 16,876 patients from 1984 to 2018. The data concerning visit’s free-form text were enriched by a subset of the hospital discharge registry of the Veneto Region, an administrative claims database, limited to the data of 16,292 patients with diabetes who were treated at the University Hospital of Padova from 2011 to 2018. The study was conducted in accordance with the principles of the Declaration of Helsinki. In compliance with national regulations on retrospective studies using routinely accumulated data (Italian Medicines Agency, “Agenzia Italiana del Farmaco”, determination 20/03/2008), the study protocol was approved by the ethical committee of the University Hospital of Padova (prot. 75856 dated 18/12/2019) and a protocol-specific consent was waived. All patients had provided informed consent to the re-use of medical data for research purposes as a prerequisite for entering the databases.

As part of the data enrichment process, the two datasets were harmonised according to the following criteria.

- The observation period spanned from January 1st, 2011, to September 30th, 2018, i.e., the overlapping time frame between the two datasets.
- Only Italian citizens, registered as healthcare beneficiaries in the Veneto Region were considered for the analysis, to avoid false negative outcomes involving patients from neighbouring regions who visited the University Hospital of Padova only for routine check-ups, but whose other healthcare needs were met in their region of origin.
- For similar reasons, visits were considered only if they happened during the patients’ healthcare eligibility periods within the Veneto Region.
- Finally, to avoid sporadic entries, only patients with at least one visit per year in three different years were included in the analysis.

After the harmonisation step, visits and hospitalisations related to CVDs were linked to form input-label pairs. Hospitalisations for CVDs and their discharge dates were identified using ICD-9-CM diagnosis codes^21^ from 390 to 459, or ICD-9-CM intervention codes denoting revascularisation procedures (00.61–66, 36.03, 36.06–07, 36.10–19, 00.55, 39.50, 39.52, 38.48, 39.71, 39.90). For each subject and each visit recorded in the database of the diabetes outpatient clinic, the existence of a CVD hospitalisation discharge was searched back in time using the regional hospitalisation discharge registry. This process was repeated four times by considering four different time windows associated to as many clinically relevant applications (see the “Introduction” section for more details on each time window and its associated application). As a result, four distinct datasets were obtained from this linking process, each one characterised by a different length of the time window used to search for a hospitalisation back in time. Specifically, hospitalisations were first searched with an infinite time window ending on the visit’s date. All visits with a prior CVD hospitalisation were labelled with a 1, regardless of time distance. Then, CVD hospitalisation discharges were searched within an increasingly narrow window (24, 12, or 6 months) before the date of the visit. If a hospitalisation was found, the visit was labelled with a “1”, meaning that a hospitalisation preceded the visit by, at most, the window’s time width; otherwise, the visit was labelled with a “0”, i.e., there was no record of a prior hospitalisation within the given window. Initial visits with incomplete windows (i.e., such that the subject was not observed for the entire 24-, 12-, or 6-month duration prior to the visit), were removed.

To offer an alternative perspective to the natural by-visit scenario described above, for performance evaluation only, by-patient versions of the four datasets were also produced by aggregating the ground truth on a patient-by-patient basis. In practice, patients were assigned a positive label (1) if at least one of their visits was labelled with a “1”, and a negative label (0) otherwise. This process led to a simpler, but nonetheless interesting, perspective characterised by a loss of temporal resolution (any hospitalisation in the patient’s history works), but decreased chance of false negatives (at least one meaningful visit is enough) relative to the by-visit setting. Hence, whereas the by-visit setting considered each visit independently, to use all the available information for model training, in the by-patient setting, the task was only to distinguish between patients with and without previous history of CVD hospitalisations within the appropriate time window, a problem of great interest for clinicians who may want to identify patients with a past CVD hospitalisation easily and automatically instead of reading each visit’s text.

For each of the four considered time windows (infinite, 24, 12, and 6 months) the corresponding independent dataset was divided in three subsets: a training set (~ 80% of the total sample size), a validation set (10%), and a test set (10%). To avoid information leakage, all the visits belonging to the same patient were part of the same subset. The by-patient versions of the dataset comprised the same patients as their respective by-visit counterparts.

The four independent datasets were then pre-processed according to the following steps, typically used in NLP^22,23^.

- Deletion of Italian stop words (e.g., definite and indefinite articles, prepositions).
- Word stemming (inflected words are substituted by their common root).
- Deletion of the 1% least frequent words.
- Exclusion of visits consisting of less than 3 words.

Table 1 shows some relevant characteristics of the 4 versions of the dataset and their subsets (training, validation, and test) obtained after the harmonisation, linking, and pre-processing steps.

**Table 1:** Dataset characteristics.

Time window	Subset	N. patients	N. visits	N. positive visits
Infinite	Training	5056	55,765	1940 (3.5%)
	Validation	632	7346	252 (3.4%)
	Test	632	6760	231 (3.4%)
	Total	6320	69,871	2423 (3.5%)
24 months	Training	5073	58,450	1935 (3.3%)
	Validation	634	7119	229 (3.2%)
	Test	635	7119	231 (3.2%)
	Total	6342	72,688	2395 (3.3%)
12 months	Training	5100	60,686	1836 (3.0%)
	Validation	638	7712	239 (3.1%)
	Test	638	7433	230 (3.1%)
	Total	6376	75,831	2305 (3.0%)
6 months	Training	5123	62,554	1632 (2.6%)
	Validation	641	7740	205 (2.6%)
	Test	640	7568	198 (2.6%)
	Total	6404	77,862	2035 (2.6%)

Dataset characteristics: number of patients included in each data subset, total number of visits, and number of positive visits. Details of the 4 versions of the dataset are reported independently while also considering the training/validation/test subset splits. Frequencies of positive visits are reported within round brackets in the last column.

### Model architecture and hyperparameters optimisation

**Figure 1:**
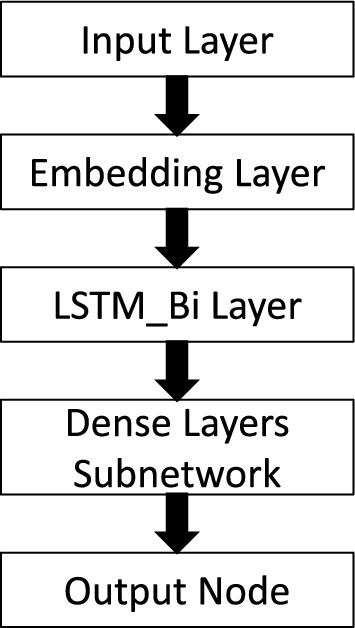
Network architecture characterised by the input layer followed by an embedding layer and a bidirectional LSTM layer. The network ends with a subnetwork of dense layers progressively halving in size before converging into a single output node. The optimal layer dimensions obtained from the hyperparameters optimisation step are reported in Table 2 for all considered time windows.

In this study, bidirectional long short-term memory (LSTM) neural networks^24^ were preferred to other DL architectures or more traditional ML methods based on bag-of-words or paragraph vectors as their performance proved to be superior in similar NLP applications^13^. More complex architectures, such as BERT, were not considered in the present study as they have been proved to work very well with English text, but it is unclear that they retain the same level of flexibility and performance when dealing with the Italian language^25^. The network was developed to identify the occurrence of a CVD hospitalisation prior to each visit. Its architecture, shown in Fig. 1, was a cascade of an embedding layer^26^, a bidirectional LSTM layer with tanh (output) and sigmoid (recurrent) activation functions^27^, and a subnetwork of dense layers with ReLU activations ending in a single output neuron with sigmoid activation (hospitalisation vs. no hospitalisation prior to the visit).

The hyper-parameters that were considered for the optimisation step were: the dimension of the embedding layer (32, 64, 128, or 256), the dimension of the LSTM layer (16, 32, 64, or 128), the non-recurrent dropout rate of the LSTM layer (0, 0.15, or 0.3), the number of dense layers (2 to 6) and dimension of the first and largest one (16, 32, 64, or 128; with each following layer in the subnetwork being half as large as the one immediately preceding it), and the dropout rate of dense layers (no dropout, or 0.1 for all dense layers).

Hyperparameter optimisation was performed on the training set via fivefold cross validation^28^ and a random search approach^29^ considering 200 combinations. The best combination of hyperparameters was selected as the one that led to the minimum average binary cross-entropy loss across the fivefold. Adam was used as optimisation algorithm for network training^30^, and the initial learning rate was set to $5\times {10}^{-5}$ with a decay rate equal to the initial learning rate divided by the maximum number of epochs (200). An early stopping criterion was considered to avoid overfitting while reducing training time: the training process was stopped after 20 consecutive epochs with no improvement^31^.

After this first optimisation step, the model with optimal hyperparameters was re-trained on the whole training set. The re-training process was repeated 100 times starting from different, randomised initialisations, and the best performing model according to the binary cross-entropy loss on the validation set was selected as the final model.

The output of the model, to be compared to the binary ground truth denoting presence or absence of CVD hospitalisation prior to each visit, was a scalar, continuous quantity between 0 and 1. To further obtain an operating point for the model, i.e., to turn it from a ranker into a proper classifier, readily useable for CVD identification, two thresholding schemes were implemented. The first one consisted in setting a single probability threshold ($th$) to distinguish between the positive (1, if predicted probability $p\ge th$) and negative (0, if $p<th$) model predictions; the second one in finding two thresholds, a low ($t{h}_{low}$) and high ($t{h}_{high}$) one, to distinguish between positive (1, if $p\ge t{h}_{high}$), negative (0, if $p\le t{h}_{low}$), and uncertain (− 1, if $t{h}_{low}<p<t{h}_{high}$) predictions.

Thresholds were optimised via the F1-score as it well balances precision and recall, equally relevant metrics whose individual optimisation would lead to a perfect value (1) for one metric and a poor value (< 0.2) for the other. For the single-threshold scenario, the optimal threshold was selected by computing the F1-score using each unique probability value predicted for visits in the validation set as a cut-off and choosing the one that led to the maximum F1-score. For the double threshold scenario, 4 different target uncertainty levels were set, namely: 5%, 10%, 15%, and 20%. For each level of uncertainty, the corresponding two optimal thresholds were identified on the validation set among 500,000 possible combinations. The best threshold combination was chosen as the one that led to the highest F1-score while excluding a fraction of patients as close as possible to the target uncertainty level, thus minimising the following cost function ${J}_{th}$:

1 $${J}_{th}=\left|F{1}_{th}-1\right|+\left|{U}_{th}-U\right|,$$

where $F{1}_{th}$ is the F1-score, ${U}_{th}$ is the uncertainty level, and $U \in ({0.05,0.1,0.15,0.2})$ is the target uncertainty level. Intuitively, setting a level of uncertainty corresponds to ignoring model predictions for the uncertain subset of visits, so that they can, e.g., be evaluated manually after the application of the model (which may be preferable to trusting predictions that are known to be unreliable).

Four different neural networks and their thresholds were optimised independently, once for each considered time window (infinite, 24, 12, and 6 months).

### Performance evaluation

When dealing with high imbalance between the positive and negative classes (only ~ 4% of visits were positive, as per Table 1) one may try to produce balanced versions of the data or use specific weighted cost functions to train the models. However, these approaches proved to be unsuccessful in improving models developed within this study. Therefore, the discrimination performance of the model was evaluated on the by-visit test set via four complementary metrics that provide a broad evaluation of discrimination while allowing the identification of data imbalance-related issues. Specifically, the considered metrics were the area under the precision-recall curve (AUPRC)^32^ for the continuous output of the network; and precision, recall, and F1-score after thresholding. In the by-patient setting, the AUPRC was not considered as predicted labels were assigned by aggregating by-visit outputs after thresholding with an OR operation.

When uncertainty was considered, visits deemed to be uncertain by the models were excluded from performance metrics computation both in the by-visit and in the by-patient setting. However, in the by-patient setting, the exclusion of uncertain visits rarely resulted in the exclusion of patients as, for the majority of them, visits classified as uncertain were only a minor portion of all their visits.

## Results

### Pre-processing and hyperparameters optimisation results

**Figure 2:**
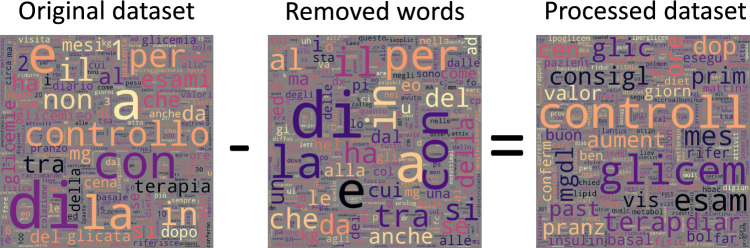
Word clouds of the original dataset (left), removed words (middle), and processed dataset (right). The removed words are Italian stop words and 1% least frequent words. Words in the processed dataset are stemmed.

Figure 2 shows the word clouds obtained from the original dataset (left), the words removed from the pre-processing steps (middle), and the final version of the dataset (right) to visualise how the processed dataset was obtained by word stemming, removing Italian stop words and least frequent words from the original corpus.

Table 2 shows the optimal hyper-parameters of the network architectures for the four different scenarios. Interestingly, the architecture found for the 12-month case was also optimal for the 24-month one. When considering a 6-month window, the optimal architectures had bigger mbedding layers (embedding dimension: 256 vs. 64) but fewer (dense layers number: 5 vs. 4 vs. 2) and maller (dense layer max dimension: 128 vs. 64 vs. 16) dense layers. The optimal LSTM dropout rate was lower for the 12- and 24-month window (0.15 vs. 0.3 in all other cases) and never equal to 0. The optimal LSTM layer dimension was 128 (effectively 256 as the LSTM layer is bidirectional) for all considered windows. Finally, the optimal dropout rate of dense layers was 0 for all versions of the dataset, suggesting that the regularisation effect was already covered by the implementation of an early stopping criterion.

**Table 2:** Optimal hyperparameters at different time windows.

Time window	Embedding dimension	LSTM dimension	LSTM dropout	Dense layers number	Dense layers dimension	Dense layers dropout
Infinite	64	128	0.3	5	128	0
24 months	64	128	0.15	4	64	0
12 months	64	128	0.15	4	64	0
6 months	256	128	0.3	2	16	0

Optimal hyperparameters obtained by minimising the cross validation binary cross-entropy loss.

### Classification results: by-visit setting

**Figure 3:**
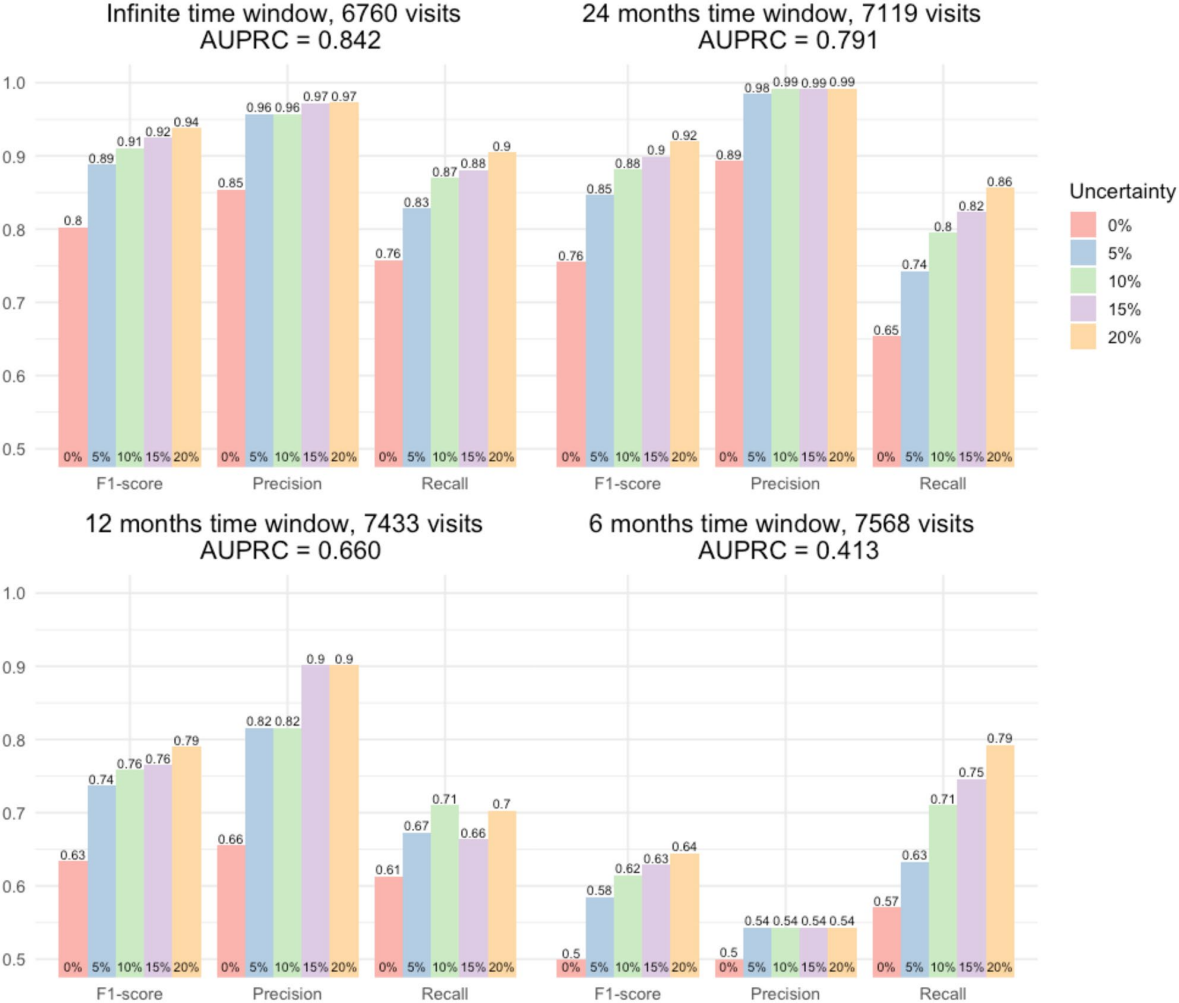
By-visit performance evaluation metrics computed on the test set for the 4 considered time windows. The number of visits that characterises each dataset is reported next to each window name in each panel title. Bars are color-coded according to the percentage of visits in the test set classified as uncertain when using two thresholds.

Figure 3 shows the performance metrics obtained in the by-visit setting, where every visit was considered independently of the others. Each panel of Fig. 3 shows, F1-score, precision, and recall reported considering different levels of uncertainty. The red bar refers to the single threshold scheme (0% uncertainty) meanwhile blue, green, violet, and orange bars relate to the double threshold scheme with target uncertainty levels 5%, 10%, 15%, and 20% respectively.

The best results were obtained when using an infinite time window (Fig. 3, top-left panel). With a single threshold (0% uncertainty) results were good (F1-score = 0.803) and, with two thresholds, it was possible to achieve very good results even with a low uncertainty level (F1-score = 0.888 with 5% uncertainty). Excellent results could be obtained by further increasing the uncertainty level (F1-score = 0.938 with 20% uncertainty). AUPRC was best in this scenario as well (0.842).

With a 24-month time window (Fig. 3, top-right panel), and a single threshold (0% uncertainty) precision was good (0.893), but recall was low (0.654). With two thresholds it was possible to achieve very good results even with a low uncertainty level (F1-score = 0.847 with 5% uncertainty). Excellent results could be obtained by further increasing the uncertainty level (F1-score = 0.920 with 20% uncertainty). In this scenario AUPRC was also acceptable (0.791).

Results worsened when considering shorter time windows. With a 12-month window (Fig. 3, bottom-left panel) and a single threshold (0% uncertainty) results were not acceptable (F1-score = 0.634); however, the double threshold scheme led to acceptable results with a moderate uncertainty level (F1-score = 0.759 with 10% uncertainty). AUPRC was not satisfactory in this scenario (0.660).

Finally, with a 6-month window (Fig. 3, bottom-right panel) and a single threshold (0% uncertainty), results were again not acceptable (F1-score = 0.499). Using a double threshold approach in this scenario was not sufficient to obtain acceptable results as the maximum F1-score was only 0.645 despite the exclusion of 20% of visits classified as uncertain. The AUPRC was not acceptable either (0.413).

The unsatisfactory results obtained in the 12- and 6-month scenarios were mainly due to the high number of false positives. This was expected, as the likelihood of encountering a visit that mentions CVD but finding no corresponding hospitalisation increases as the window gets narrower, mainly owing to the relatively high proportion of patients who schedule routine visits at > 1-year intervals.

### Classification results: by-patient setting

**Figure 4:**
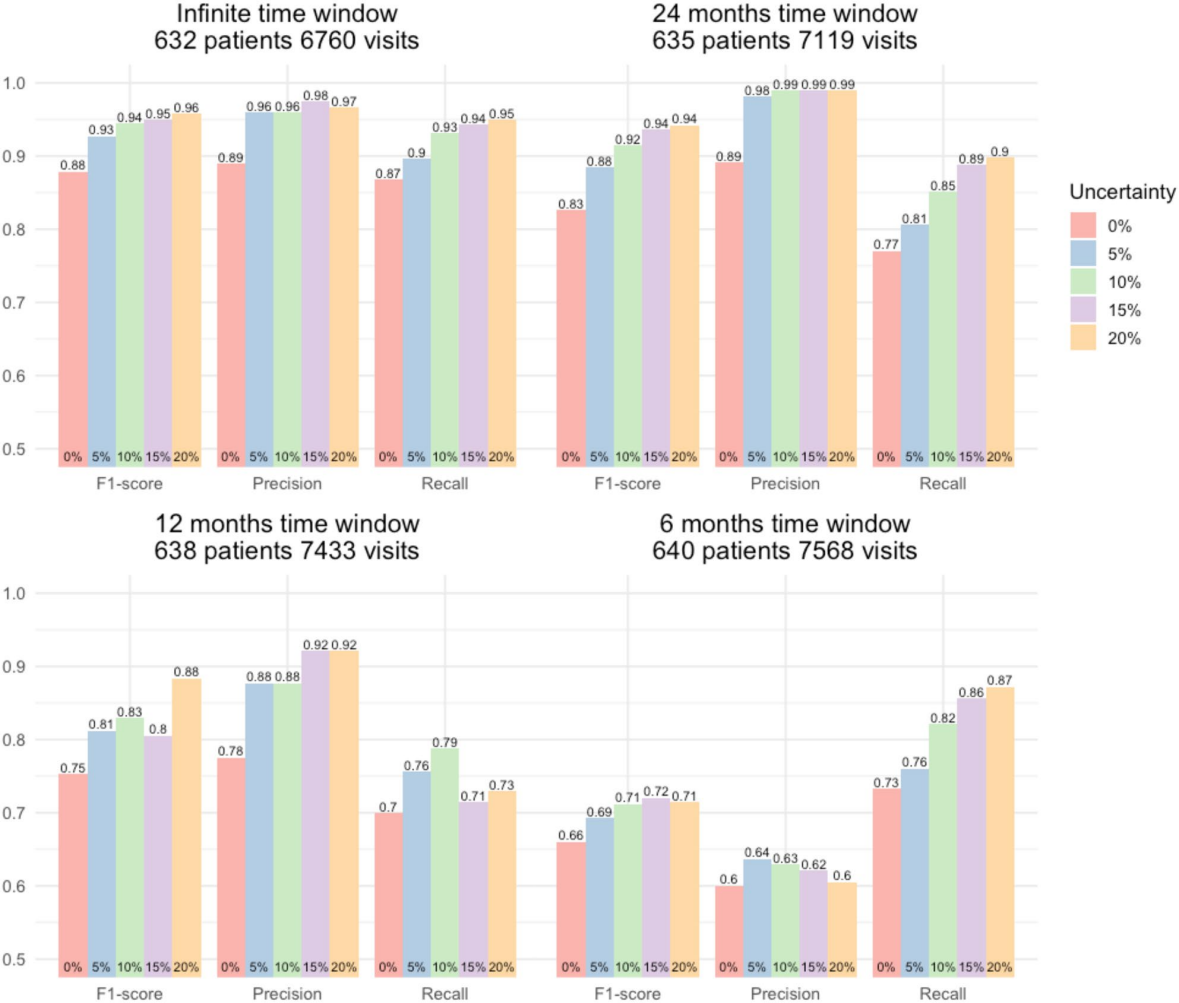
By-patient performance evaluation metrics computed on the test set for the 4 considered time windows. The number of patients that characterises each dataset is reported in each panel title. Bars are color-coded according to the percentage of visits in the test set classified as uncertain when using two thresholds.

Figure 4 shows the performance metrics considered in the by-patient setting, where visits were grouped according to the patients they belonged to. For each window width, performance metrics were evaluated on the test sets: infinite (Fig. 4, top-left panel), 24 months (top-right panel), 12 months (bottom-left panel), and 6 months (bottom-right panel). Each panel of Fig. 3 shows F1-score, precision, and recall reported considering different levels of uncertainty. The red bar refers to the single threshold scheme (0% uncertainty) meanwhile blue, green, violet, and orange bars relate to the double threshold scheme with target uncertainty levels 5%, 10%, 15%, and 20% set at the visit level. AUPRC was not considered in the by-patient setting as explained in “Performance evaluation” section.

The best results were obtained when considering an infinite time window (Fig. 4, top-left panel). With a single threshold (0% uncertainty) results were good (F1-score = 0.879) and by considering a double threshold approach it was possible to achieve very good results even with a low uncertainty level (F1-score = 0.927 with 5% uncertainty). Excellent results could be obtained by further increasing the uncertainty level (F1-score = 0.958 with 20% uncertainty).

With a 24-month window (Fig. 4, top-right panel), and a single threshold (0% uncertainty) results were still good (F1-score = 0.826) and by considering two thresholds it was possible to achieve very good results even with a low uncertainty level (F1-score = 0.885 with 5% uncertainty). Excellent results could be obtained by further increasing the uncertainty level (F1-score = 0.942 with 20% uncertainty).

With a 12-month window (Fig. 4, bottom-left panel) and a single threshold (0% uncertainty) results were not acceptable as precision was good (0.775), but recall was low (0.699). Considering a double threshold approach led to acceptable results even with a low uncertainty level (recall = 0.756 with 5% uncertainty). Better results were possible by increasing the uncertainty level (F1-score = 0.884 with 20% uncertainty).

With a 6-month window (Fig. 4, top-left panel) and a single threshold (0% uncertainty) results were not acceptable (F1-score = 0.660); despite the model achieving good recall (0.733), precision was low (0.600) Precision remained low (max 0.637) even when using two thresholds.

While not directly comparable, performance was overall better in the by-patient setting than in the by-visit one, which was expected as it is easier to obtain a correct classification look at multiple visits for each patient rather than classifying each visit independently.

Despite a high level of uncertainty at the visit level (~ 20%), at the patient level few patients were excluded for having all visits classified as uncertain (4–6). These results justify the use of a two-threshold scheme and a relatively low uncertainty level (5–10%), especially for the by-patient setting.

When considering infinite, 24-, and 12-month windows, false negatives (rather than false positives) were the main drivers of performance degradation. As a positive label is associated to a visit close to a CVD hospitalisation, false negatives, in this context, consist of visits that are close to CVD hospitalisations but are not recognised as such based on their free-form text. The higher number of false negatives is, thus, mostly due to how the clinicians record relevant information in the free-form text. Specifically, in the text of positive visits, there were mentions of CVD pathologies, hospital admission, or hospital discharge; however, this information was not always present, e.g., because the specialist may not have discussed previous hospitalisations with the patient, but only their general health status, glycaemic control, or diet. Hence, even an expert would not have been able to classify these particular visits correctly based on free-form text alone.

When using two thresholds, recall tended to increase more sharply than precision even with a low uncertainty level. The main drivers of this result were once again those false negatives given by positive visits characterised by free-form text with no mention of CVD. In a two-threshold scenario, the vast majority of these visits ended up being classified as uncertain and thus excluded from the computation of performance metrics, leading to the observed sharp increase in recall.

## Conclusions

In this study, a set of neural networks was developed to associate free-form text written by clinicians during the routine visits of patients with diabetes to previous CVD hospitalisations within different time windows. To this end, a specialist-care database was enriched with the hospitalisations records retrievable from the local administrative claims repository (N ~ 6400 unique, harmonised patients).

Four different time windows were considered when looking for hospitalisations prior to each visit: infinite, 24, 12, and 6 months. Results obtained with the first two windows, suggest that the proposed NLP model could be reliably used to automatically fill patients’ medical records or identify recent CVD events. Moreover, in these scenarios, discrimination performance could be remarkably improved with a limited workload from clinicians (as few as 328 visits to be manually parsed to obtain an F1-score of 0.847). Not surprisingly, shortening the time window produces a deterioration of the discrimination performance, in fact, with a 12-month window, satisfactory results could not be obtained without assuming a significant contribution by clinicians (upwards to 736 visits to assess manually to obtain an F1-score of 0.759). In the 6-month window scenario, the discrimination performance was once more not acceptable even when allowing for a contribution by clinicians. This suggests that it is not possible to use the proposed approach to translate routine visits’ free-form text into a CVD hospitalisation time-to-event outcome.

Tools based on these approaches may be useful for clinicians as they may help address the known problem that, when faced with a choice between reporting information in a structured or an unstructured field, physicians tend to prefer the latter, which is more in line with their attitude and training. Case in point, in the eCharts used in this study, among patients who had a CVD hospital discharge, only one in three had previous history of CVD correctly reported in the structured section of their healthcare record, despite almost all having at least one visit with mentions of hospitalisations or CVD. Moreover, NLP tools that automatically read all of a patient’s visits may help overcoming the fatigue clinicians may encounter in re-reading bulks of text buried in different records of a patient’s EHR to recall their history of prior CVD. Payers and administrators may also benefit from the use of such tools as they could better investigate whether relevant information, such as previous pathologies, are coherently reported in EHR systems, and possibly implement mitigation strategies, e.g., if they are not satisfied with the reporting rate.

Future studies may focus on the development of more complex architectures or training schemes, with the aim of improving performance on shorter time windows, i.e., when positive visits are rare, and models struggle to successfully learn key features useful to distinguish them from negative visits. Use of an external dataset, presently not available, would help in validating the method against more heterogeneous data, with visits coming from different clinics, where clinicians may follow slightly different protocols or conventions.

## Data Availability

The datasets generated during and/or analysed during the current study are not publicly available as they are owned by the Regional Healthcare System and were used under license for the current study. Data are however available from the authors upon reasonable request and with permission of the Regional healthcare system.
